# Molecular mechanism of modulating miR482b level in tomato with *botrytis cinerea* infection

**DOI:** 10.1186/s12870-021-03203-2

**Published:** 2021-10-28

**Authors:** Fangli Wu, Jinfeng Xu, Tiantian Gao, Diao Huang, Weibo Jin

**Affiliations:** Key Laboratory of Plant Secondary Metabolism and Regulation of Zhejiang Province, College of Life Sciences and medicine, Zhejiang Sci-Tech University, 310018 Hangzhou, China

**Keywords:** sly-miR482b, pri-miR482b isoforms, posttranscriptional processing, pathogen response, *Botrytis cinerea*

## Abstract

**Background:**

Plant miRNAs are involved in the response to biotic and abiotic stresses by altering their expression levels, and they play an important role in the regulation of plant resistance to stress. However, the molecular mechanism that regulates the expression levels of miRNAs in plants with biotic and abiotic stress still needs to be explored. Previously, we found that the expression of the miR482 family was changed in tomato infected by *Botrytis cinerea*. In this study, we investigated and uncovered the mechanism underlying the response of miR482 to *B. cinerea* infection in tomato.

**Results:**

First, RT-qPCR was employed to detect the expression patterns of miR482b in tomato infected by *B. cinerea*, and results showed that miR482b primary transcripts (pri-miR482b) were up-regulated in *B. cinerea*-infected leaves, but the mature miR482b was down-regulated. Subsequently, we used rapid amplification cDNA end method to amplify the full-length of pri-miR482b. Result showed that the pri-miR482b had two isoforms, with the longer one (consisting 300 bp) having an extra fragment of 53 bp in the 3’-end compared with the shorter one. In vitro Dicer assay indicated that the longer isoform pri-miR482b-x1 had higher efficiency in the post-transcriptional splicing of miRNA than the shorter isoform pri-miR482b-x2. In addition, the transcription level of mature miR482b was much higher in transgenic *Arabidopsis* overexpressing pri-miR482b-x1 than that in OE pri-miR482b-x2 *Arabidopsis*. These results confirmed that this extra 53 bp in pri-miR482b-x1 might play a key role in the miR482b biogenesis of post-transcription processing.

**Conclusions:**

Extra 53 bp in pri-miR482b-x1 enhanced miR482b biogenesis, which elevated the transcription level of miR482b. This study clarified the response of miR482 to *B. cinerea* infection in tomato, thereby helping us further understand the molecular mechanisms that regulate the expression levels of other miRNAs.

**Supplementary Information:**

The online version contains supplementary material available at 10.1186/s12870-021-03203-2.

## Background

MicroRNAs (miRNAs) are a class of sRNAs with length of 20 ~ 24 nt. In plants, the miRNA gene is transcribed by RNA polymerase II [1]. The primary transcripts (pri-miRNAs) fold into an incomplete stem-loop structure, which is cleaved by RNase III type enzyme Dicer-like 1 (DCL1) to form an incomplete hairpin precursor miRNA, termed pre-miRNAs. The pre-miRNAs are then cleaved by DCL1 or DCL4 [2, 3] to generate the miRNA duplex, namely miRNA: miRNA* double-stranded dimer. Methylation of the 3’-end of the duplex is by methyltransferase HEN1. The plant exportin-5 homologous protein (HASTY, HST) transports the miRNA duplex into the cytoplasm, and they are incorporated into the RISC (RNA-induced silencing complex) together with an Argonaute (AGO) protein, where one strand is selected to become the mature miRNA [4], binding to the target mRNA complements to cleave or inhibit translation to achieve negative regulation of the target gene. In addition to the above classical mode of miRNA synthesis, it can be synthesized by the action of the DCL enzyme or the like [5].

MiRNAs act as negative regulators of gene expression in eukaryotes and participate to regulate the growth and development of plants [6–10] and disease resistance [11–14]. To date, many miRNAs exhibit different expression profiles in plants responding to *B. cinerea* infection. Zhao et al. found that miR5254, miR165a-3p, miR3897-3p and miR6450a are involved in the defense response of tree peony during the invasion of *B. cinerea* [15]. Soto-Suarez et al. found that in addition to its role in controlling development, miR396 contributes to the dynamic defense response to necrotrophic (*B. cinerea*) and hemibiotrophic (*P. cucumerina*) fungal pathogens in *Arabidopsis* [16]. In strawberry, miR5290a negatively regulates its target gene PIRL to increase resistance to *B. cinerea* [17]. The 31 miRNAs in lily are differentially expressed in leaves infected by *Botrytis ellipsoidea* and respond to the stress of *B. ellipsoidea* [18]. In our previous study, we found that the expression levels of multiple miRNAs in tomato are correlated with *B. cinerea* infection via miRNA microarray and high-throughput sequencing technology [19, 20]. Moreover, miR319 and miR394 act as positive and negative regulators in resistance to *B. cinerea* infection, respectively [21, 22].

Although the expression levels and the biological functions of many miRNAs have been well studied in plants in response to environmental changes, the mechanisms that regulate the expression of the environmentally responsive miRNAs are still poorly understood. Bielewicz et al. found that introns are crucial for the expression levels of two miRNA genes: MIR163 and MIR161. Removal of their introns leads to a drop-off in the level of both miRNAs [23]. In addition, Schwab et al. showed that the introns located in the 3’ -end of the stem-loop structure can promote mature miRNA accumulation [24]. Inhibition of splicing at the 93 − 25 3’ SS (between the sequences of pre-miRNA 93 and 25) results in elevated miRNA levels [25]. In the previous study, we found that the expression level of miR482 was changed in tomato leaves with *B. cinerea* infection [26]. In the present study, we found that miR482b was significantly down-regulated in tomato leaves infected by *B. cinerea*, but its primary transcript was up-regulated. This result suggested that the different expression patterns between miR482b and its primary might be involved in the processing efficiency of post-transcriptional levels rather than that in transcription levels. Therefore, in this study, we aimed to reveal the mechanism that regulated the expression of miR482b in tomato’s response to *B. cinerea* infection.

## Results

### 1. Expression patterns of miR482b and its primary in ***B. cinerea***-infected tomato leaves

**Fig. 1:**
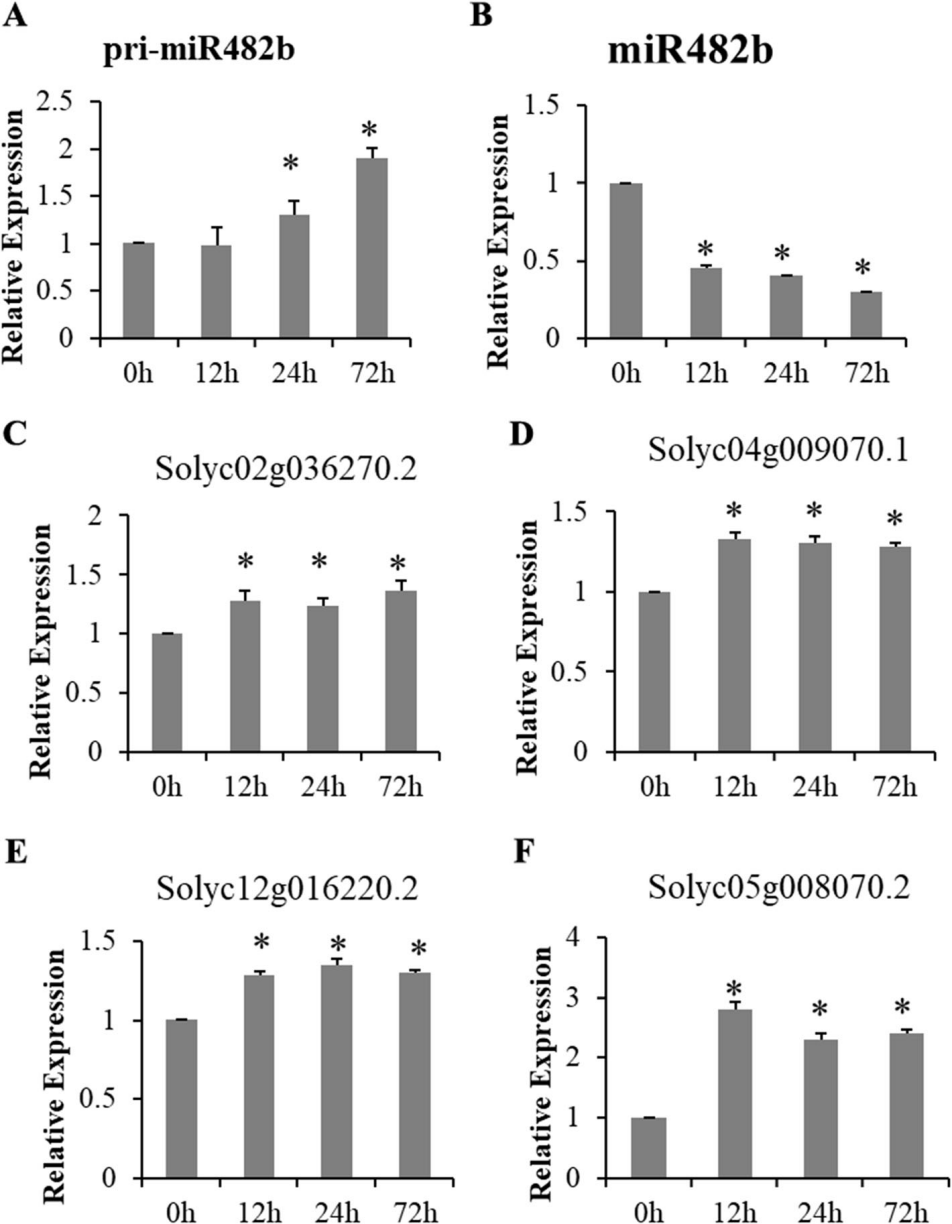
Expression patterns of miR482b and its target genes in mock- and *B. cinerea*-infected leaves at 0, 12, 24 and 72 hpi. **A-B**) The expressions of pri-miR482b (**A**) and miR482b (**B**). *SlyU6* were used as the internal control. Results are expressed as means ± SD of three biological replicates. Asterisks indicate a significant difference (*P* < 0.05) compared with the corresponding 0 hpi leaves. **C-F**) The expressions of four NBS-LRR genes (*Solyc02g036270.2, Solyc04g009070.1, Solyc12g016220.2*, and *Solyc05g008070.2*) targeted by miR482b. *SlyUbq3* were used as the internal control. Results are expressed as means ± SD of three biological replicates. Asterisks indicate a significant difference (*P* < 0.05) compared with the corresponding 0 hpi leaves

To understand the expression patterns of miR482b, the abundance of the mature miRNA and its primary (pri-miR482b) was measured by quantitative reverse transcription PCR (RT-qPCR) in *B. cinerea*-infected tomato at different time points. The results showed that the abundance of pri-miR482b was significantly up-regulated at 24 and 72 hpi (Fig. 1 A). Interestingly, the expression level of miR482b was significantly down-regulated at all three time points (Fig. 1B). Correspondingly, four nucleotide binding site-leucine-rich repeat genes (NBS-LRR), namely *Solyc02g036270.2, Solyc04g009070.1, Solyc12g016220.2*, and *Solyc05g008070.2*, which were experimentally confirmed as the target genes of miR482b [27], were up-regulated in *B. cinerea*-infected leaves at all three time points compared with 0 hpi (Fig. 1 C-F), showing negative regulation by miR482b. These results proposed that miR482b was induced at the transcriptional level but inhibited the splicing process after transcription in tomato leaves infected by *B. cinerea.*

## 2. Cloning and chromosomal location of pri-miR482b

**Fig. 2:**
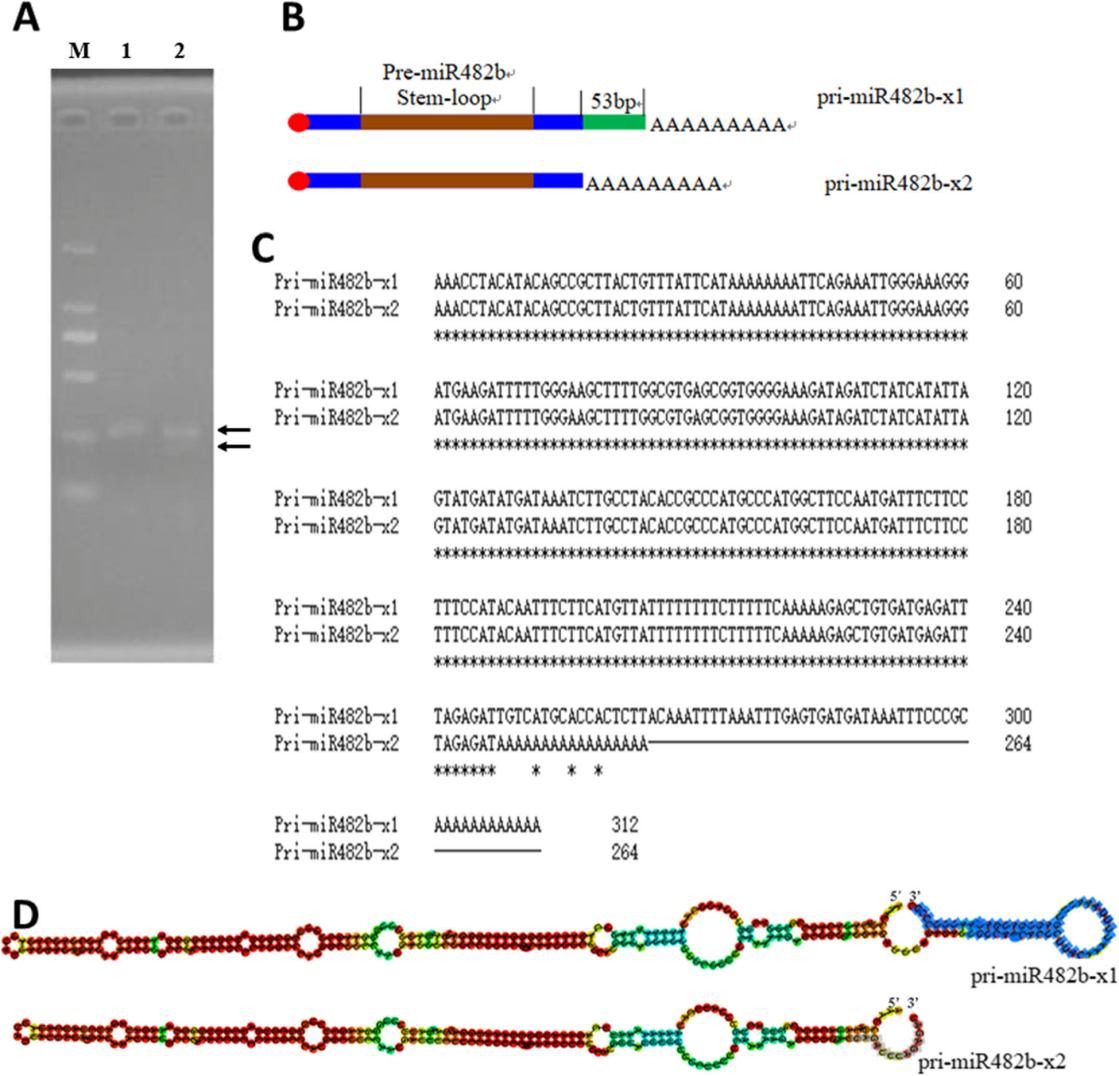
Cloning and sequence analysis of full-length pri-miR482b. **A**) RACE Amplification of pri-miR482b. Lane M: DL 2000 DNA marker; Lane 1: 5’ end of pri-miR482b; Lane2: 3’ end of pri-miR482b. **B**) Schematic of two isoforms of pri-miR482b. **C**) Alignment of two isoforms of pri-miR482b. **D**) Secondary structures of two pri-miR482b isoforms

To detect whether the down-regulation of miR482b is due to post-transcriptional inhibition, the full-length cDNA of the miR482b primary was first cloned by the RACE method. The results showed that a 256 bp fragment was amplified by 5’-RACE (Fig. 2 A). Interestingly, 3’-RACE results showed two different amplicons in 3’-RACE (Fig. 2 A). DNA sequencing revealed that the longer one was 254 bp in length, and the shorter one was 201 bp, with a 53 bp deletion at the 3’-end of the longer one. The complete cDNA of the miR482b gene was compiled by overlapping the sequences of the cloned cDNA and the 5’-RACE and 3’-RACE PCR products. Two isoforms of pri-miR482b transcripts without poly(A) consisted of 300 and 247 bp, respectively. The longer isoform was named pri-miR482b-x1, and the shorter one was named pri-miR482b-x2 (Fig. 2B C). Compared with pri-miR482b-x2, an extra small hairpin structure was presented in the 3’-end of pri-miR482b-x1 (Fig. 2D). On the basis of the full-length cDNA sequence of pri-miR482b, miR482b was located in the intergenic region, and the transcription start site was at 37,497,067 of the minus strand of the tomato chromosome 6 which had a full length of 49,794,276 bp. Both sequences have been deposited into the GenBank database (accession numbers: MW590251 and MW590252).

### 3. Expression patterns of pri-miR482b-x1 and pri-miR482b-x2 in ***B. cinerea***-infected tomato leaves

**Fig. 3:**
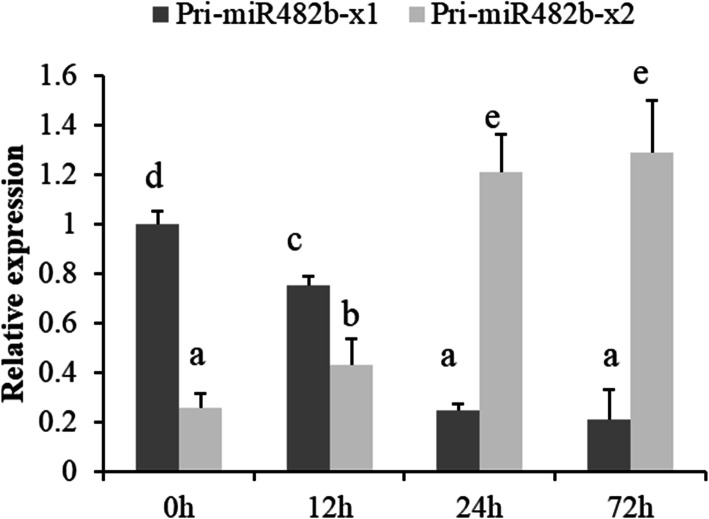
Expression patterns of two pri-miR482b isoforms in mock- and *B. cinerea*-infected leaves at 0, 12, 24 and 72 hpi. *SlyUbq3* was used as the internal control. Results are expressed as means ± SD of three biological replicates. Various letters indicate a significant difference among samples (*P* < 0.05)

To understand the expression patterns of both isoforms of pri-miR482b, the abundance of pri-miR482b-x1 and pri-miR482b-x2 was further quantified in *B. cinerea*-infected tomato at different time points. The transcript level of pri-miR482b-x1 was significantly down-regulated in *B. cinerea*-inoculated leaves at 12, 24 and 72 hpi compared with that at 0 hpi, whereas the transcript level of pri-miR482b-x2 was significantly up-regulated at 24 and 72 hpi (Fig. 3). These results indicated that miR482b was down-regulated in *B. cinerea*-inoculated leaves due to the inhibited expression of pri-miR482b-x1. Thus, the extra stem-loop structure in pri-miR482b-x1 might play a key role in the miR482b biogenesis of post-transcription processing.

### 4. Detection of the splicing efficiency for the two pri-miR482b isoforms

**Fig. 4:**
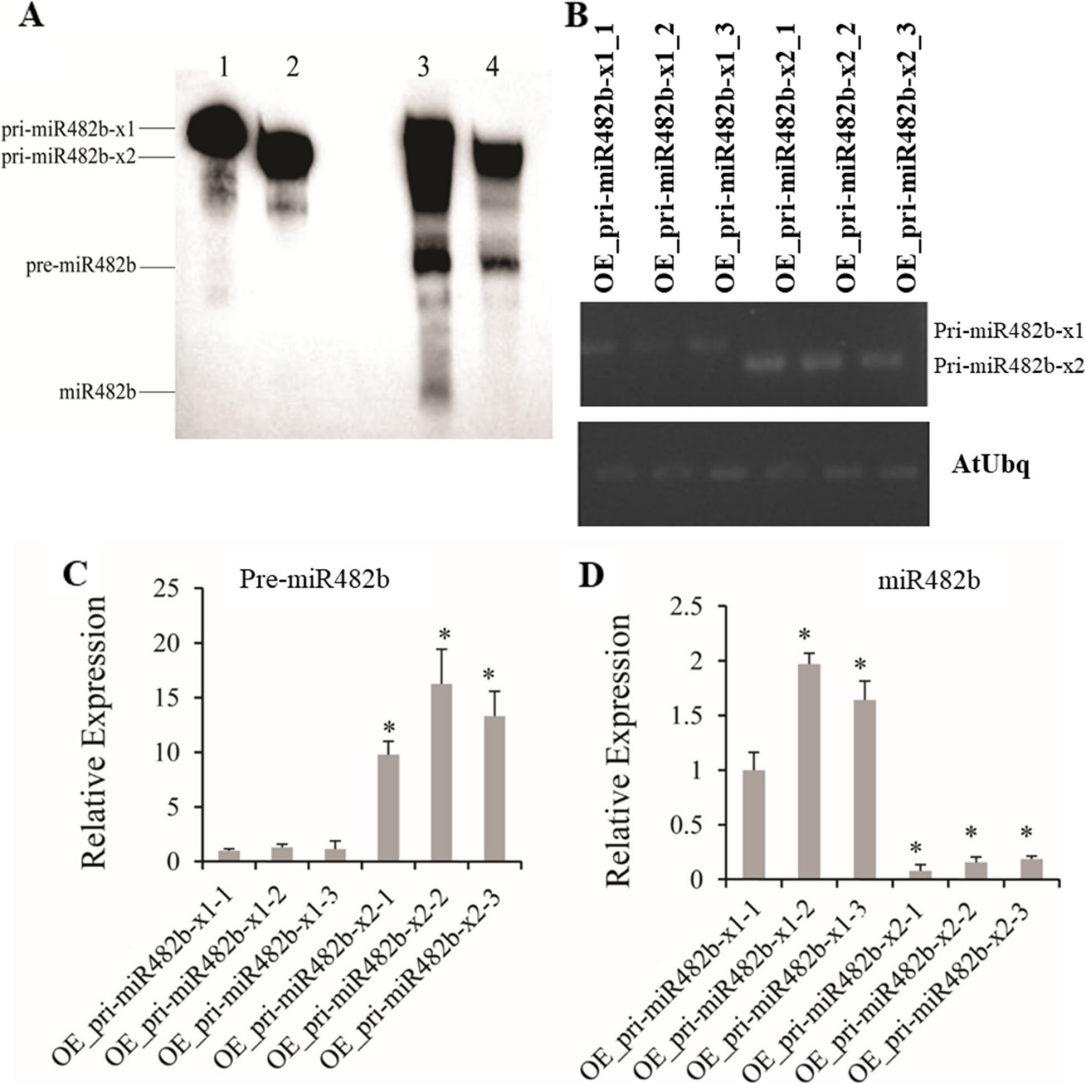
Dicer splicing activities of two pri-miR482b isoforms. (**A**) Splicing efficiencies of two pri-miR482b isoforms in vitro. Land 1: pri-miR482b-x1; Lane 2: pri-miR482b-x2; Lane 3: pri-miR482b-x1 with 2 mg/mL protein extraction; Lane 4: pri-miR482b-x2 with 2 mg/mL protein extraction. (**B**) Expression levels of pri-miR482b-x1 in pri-miR482b-x1 OE plants and the expression level of pri-miR482b-x2 in pri-miR482b-x2 OE plants via semi-quantification RT-PCR. Lanes 1–3: three OE lines of pri-miR482b-x1; Lanes 4–6: three OE lines of pri-miR482b-x2. **C-D**) Expression levels of pre-miR482b (**C**) and miR482b (**D**) in pri-miR482b-x1 and pri-miR482b-x2 OE plants via quantification RT-PCR. *AtUBQ10* was used as the internal control. Results are expressed as means ± SD of three biological replicates. Asterisks indicates a significant difference (*P* < 0.05) compared with the corresponding OE_pri-miR482b-x1-1

To detect whether the two isoforms of pri-miR482b have different efficiencies in the biogenesis of miR482b, both biotin-labeled pri-miR482b-x1 and -x2 were transcribed in vitro via T7 RNA polymerase with NTPs and biotin-labeled UTP and then incubated with 2 µL of miRNA splicing proteins (5 mg/mL). The results showed that pri-miR482b-x1 was spliced to produce pre-miR482b-x1 and mature miR482b, whereas pri-miR482b-x2 only produced a pre-miR482b-like fragment; no mature miR482b was produced (Fig. 4 A; Additional file 1 (Figure S1B-C)).

To investigate the splicing efficiency of both isoforms in vivo, *Arabidopsis*, which does not have an endogenous miR482 gene was selected for overexpressing pri-miR482b-x1 and pri-miR482b-x2 by transgenic technology. RT-PCR results showed that pri-miR482b-x1 and -x2 were expressed in transgenic plants overexpressing pri-miR482b-x1 and -x2, respectively. The expression levels of pri-miR482b were lower in pri-miR482b-x1 overexpression (OE) plants than in pri-miR482b-x2 OE plant (Fig. 4B). Moreover, RT-qPCR results revealed that the expression level of pre-miR482b was lower in pri-miR482b-x1 OE plants than in pri-miR482b-x2 OE plants (Fig. 4 C), but mature miR482b had higher abundance in pri-miR482b-x1 OE plants than in pri-miR482b-x2 OE plants (Fig. 4D). These results confirmed that the 53 nt extra sequence in the 3’-end of pri-miR482b-x1 played a critical role in miR482b biogenesis after transcription.

### 5. Function of miR482b in plants infected by ***B. cinerea***

**Fig. 5:**
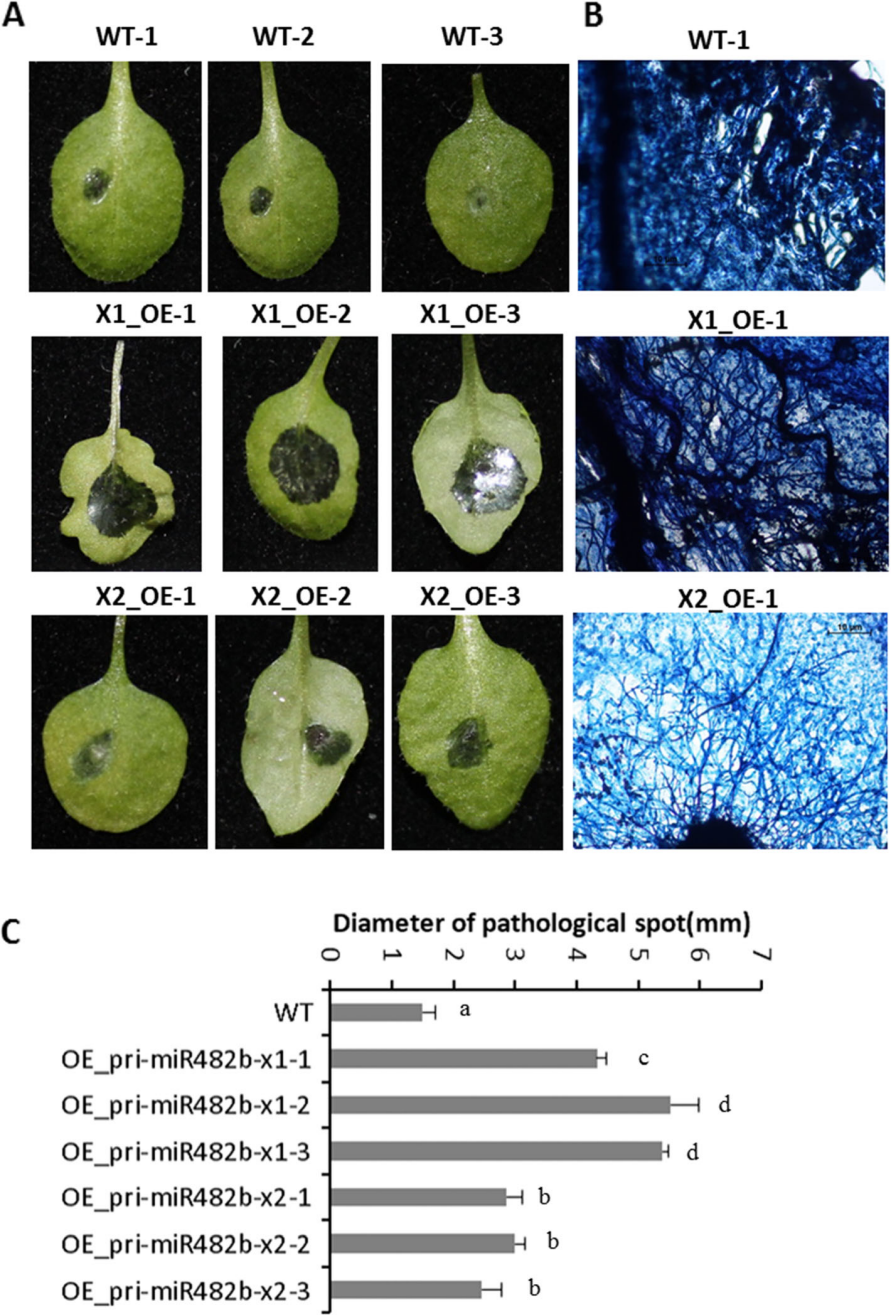
Resistance analysis of transgenic *Arabidopsis* overexpressing pri-miR482b-x1 and pri-miR482b-x2 against *B. cinerea*. (**A**) Disease symptoms on the *B. cinerea*-infected leaves of WT and transgenic *Arabidopsis* overexpressing pri-miR482b-x1and pri-miR482b-x2 for 48 h. (**B**) Trypan blue staining confirmed the pathological spots. (**C**) Statistical analysis of the pathogenic spot diameters. Asterisks indicate a significant difference (*P* < 0.01) compared with the corresponding WT

**Fig. 6:**
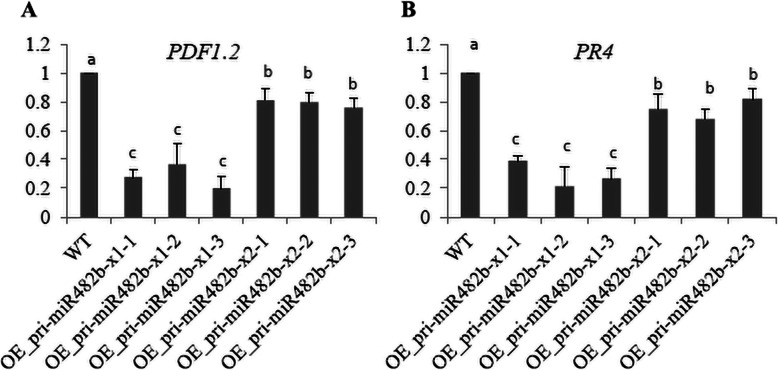
Expression levels of two genes (PDF1.2 and PR4) of JA-signal pathway in WT and transgenic *Arabidopsis*. *SlyUbq3* was used as the internal control. Results are expressed as means ± SD of three biological replicates. Various letters indicate a significant difference among samples (*P* < 0.05)

To understand the role of miR482b in plants infected by *B. cinerea*, WT and transgenic plants were inoculated with *B. cinerea* for 48 h, and the physical appearance of the plants was assessed. The transgenic plants had larger necrotic spots than the WT plants (Fig. 5 A-C; Additional file 1 (Figure S2)). Moreover, the necrotic spots of pri-miR482b-x1 OE plants were ~ 2 fold larger than those of pri-miR482b-x2 OE plants. In addition, two marker genes, plant defensing 1.2 (PDF1.2) and pathogen related protein 4 (PR4) [28], of the ERF branch of the jasmonic acid signaling pathway were quantitative detected in WT and transgenic plants. The results showed that the expression of both genes was downregulated in transgenic pri-miR482b OE plants compared with wide type plants. Moreover, the inhibition of both genes is more significant in pri-miR482b-x1 OE plants than that in pri-miR482b-x2 OE plants (Fig. 6).

## Discussion

Given the important role of miRNAs in the regulation of plant growth and development and stress response, the abundance of miRNAs is tightly regulated at multiple levels, including transcriptional and post transcriptional steps [29]. In transcriptional steps, evidence suggested that general RNA-binding proteins (RBPs) binding to the terminal loop or stem of an miRNA stem-loop structure can positively or negatively affect microprocessor mediated pri-miRNA and/or pre-miRNA processing [30]. For example, the RBP protein LIN28 can specifically recognize and bind to the terminal loop of let-7 and then affect the processing of the let-7 precursor by blocking the activity of Drosha and Dicer [31–33]. HnRNP A1 binds to the terminal loop of pri-mir-18a and induces relaxation at the stem–loop structure near the DROSHA cleavage site, resulting in increased efficiency of miRNA processing [29]. In addition, the sequence variation, such as single nucleotide polymorphisms, may play a role in the biogenesis of miRNA. A rare genetic variation in the terminal loop of pri-miR-30c-1 (G27 to A), which directly affects the processing of pri-mir-30c-1 by inducing a secondary RNA structure rearrangement and facilitates binding of the trans-acting factor SRSF3 [34], results in increased levels of mature miR-30c [34, 35]. Finally, the introns are crucial for the expression levels of two miRNA genes, namely, MIR163 and MIR161. Removal of their introns leads to a drop-off in the level of both miRNAs [23]. Schwab et al. also showed that the introns located at the 3’-end of the stem-loop can promote mature miRNA accumulation [24]. In this study, we found that the processing efficiency of pri-miR482b was inhibited when a 53 nt fragment was absent in the 3’-end of pri-miR482b.

The miR482 family comprises plant-specific small RNAs that have been found in 23 plants including *Populus trichocarpa* [36], *Pinus taeda* [37, 38], *Glycinemax* [39], *Malus domestica* [40], *Phaseolus vulgaris* [40], *Medicago truncatula* [41], *Solanum lycopersicum* [42], and so on. Evidence showed that miR482 is involved in response to biotic stress and acts as a negative regulator in plant-pathogen interaction through inhibited R genes. In tomato and potato, members of the miR482 family are expected to target approximately 20 % of resistance genes [43]. Lu et al. [36, 37] showed that Ptc-miR482 can cleave anti-pathogenic protein genes involved in plant resistance to biotic and abiotic stresses. Therefore, overexpression of miR482 can decrease the resistance of host plants against pathogens. Yang et al. [44] found that the overexpression of potato miR482e enhances the sensitivity of plants to *Verticillium dahliae* by targeting a series of NBS-LRR genes. Jiang et al. [27] found that miR482b negatively regulates the infection of *Phytophthora infestans* by targeting the NBS-LRR resistance genes. Feng et al. [45] found that ghr-miR482a plays a role in resistance to *G. barbadense* by targeting the R gene. By contrast, to increase resistance, host plants would like to reduce the expression of miR482 and increase the expression of the NBS-LRR genes in the pathogen-host interaction. Zhu et al. [46] found that the expression levels of the NBS-LRR genes were induced in *V. dahlia*-infected cottons by inhibiting the expression of ghr-miR482c, ghr-miR482d.2 and ghr-miR482b/miR482b.2. Ouyang et al. [47] found that tomato plants up-regulated an R gene by inhibiting the expression of sly-miR482f to enhance resistance to *Fusarium oxysporum*. In this study, we also found that the expression levels of miR482b and its targets were down- and up-regulated in tomato with *B. cinerea* infection, respectively, resulting in the up-regulation of its target genes (NBS-LRRs). Moreover, we confirmed that increasing the transcript level of miR482b could decrease resistance to *B. cinerea* infection in plants. This result indicated that miR482b played a negative role in plant resistance to *B. cinerea* and suggested that the plants can resist the infection of *B. cinerea* by changing the isoform levels of miR482b.

The jasmonic acid-mediated defense pathway has an important role in plant resistance against necrotrophic pathogens [48]. There are two distinct branches in *Arabidopsis* JA pathway including ethylene response factor (ERF) branch and the MYC branch [28]. The ERF branch of the JA pathway is typically activated upon infection by necrotrophic pathogens [49]. Induction of the ERF branch results in the activation of a large set of JA/ET-responsive genes, including the marker gene PDF1.2 [50, 51]. In the other branch of the JA pathway, MYC branch, is typically activated upon wounding or feeding by herbivorous insects. Therefore, we tried to understand the effect of miR482b over-expression on the JA pathway through investigating the expression of two marker genes in ERF branch of JA pathway. Results showed that the PDF1.2, a marker genes of JA pathway representative ERF branch, was significantly decreased in pri-miR482b OE plants compared to WT. Moreover, PDF1.2 has lower expression level in pri-miR482b-x1 OE plants than that in pri-miR482b-x2 OE plants (Fig. 6). On the contrary, PDF1.2 would be induced by *B. cinerea* to activate JA pathway for increasing the resistance in *Arabidopsis* [28]. In addition, a similar expression pattern was observed for PR4, which is another JA-responsive gene of the ERF branch and its expression levels could be induced by necrotrophic pathogen [52], but was also decreased in pri-miR482b OE plants compared to WT. Our results suggested that the overexpression of pri-miR482b enhanced the susceptibility of the transgenic plants to *B. cinerea* infection through inhibiting the ERF branch of JA signaling pathway.

## Conclusions

This study was the first to reveal the molecular mechanism underlying the suppression of miR482b expression in tomato infected by *B. cinerea* as follows: a 53 nt fragment was spliced in the 3’-end of the normal pri-miR482b, namely pri-miR482b-x1, in *B. cinerea*-infected tomato. The truncated pri-miR482b isoform, namely, pri-miR482b-x2, has lower processing efficiency in miR482b biogenesis, leading to the decrease in transcript level of miR482b in *B. cinerea*-infected tomato.

## Methods

### 1. Plants and ***B. cinerea*** inoculation

Seeds of tomato *cv. MicroTom*, purchased from Nanjing Fengshuo Yuanyi Co., Ltd in China. *Arabidopsis thaliana* (*Col-0*) and *Botrytis cinerea* were provided by plant pathology laboratory of Zhejiang Sci-tech University (Hangzhou, Zhejiang). Tomato and *B. cinerea* were cultured according to Meng et al. [53]. Seeds of tomato were grown with a 12:12 h photoperiod at ~ 22 °C. Six-week-old plants were inoculated with *B. cinerea* solution containing 5 × 10^6^ conidiospores/mL. The *B. cinerea*- and mock-inoculated leaves were harvested at 0, 12, 24 and 72 h post inoculation (hpi). The samples were frozen in liquid nitrogen and stored at -70 °C for transcript level analyses.

### 2. RNA extraction, reverse transcription and real-time PCR (RT-qPCR)

Total RNAs were extracted and quantified of total RNA were performed according to Meng et al. [53]. Total RNAs were extracted using TRIzol reagent, treated by RNase-free DNase, and quantified by using a NanoDrop ND-1000 spectrophotometer. For poly(A) RNAs, equal quantities of total RNA (1 µg) were reverse-transcribed at 42 °C using SuperScript III Reverse Transcriptase (Invitrogen) and 2.5 µM Oligo(dT_18_). A similar reaction without reverse transcriptase was also performed as a control to confirm the absence of genomic DNA in subsequent steps. For miRNAs, reverse transcription was performed using the One Step PrimeScript miRNA cDNA Synthesis Kit (TaKaRa, Dalian, China) according to the manufacturer’s protocol.

SYBR Green PCR was performed according to Meng et al. [53]. In brief, 2 µL of cDNA template was added to 12.5 µL of 2× SYBR Green PCR master mix (Takara), 1 µM specific primers and ddH_2_O to a final volume of 25 µL. The reactions were amplified for 10 s at 95 °C, followed by 40 cycles of 95 °C for 10 s and 60 °C for 30 s. All reactions were performed in triplicate, and controls (no template and no RT) were included for each gene. The threshold cycle (Ct) values were automatically determined by the ABI Prism 7300 Sequence Detection System (PE Applied Biosystems, USA). The fold-changes were calculated using the 2^−ΔΔCT^ method, where ΔΔCt = (Ct,target - Ct,inner)_Infection_ - (Ct,target − Ct,inner)_Mock_ [54].

### 3. Rapid amplification of cDNA ends (RACE)

The full-length of pri-miR482b was obtained by rapid amplification of cDNA ends (RACE) with a SMART RACE cDNA Amplification Kit (Invitrogen) according to the manufacturer’s protocol. Total RNA (1 µg) obtained from tomato leaves was used for cDNA synthesis. The gene-specific primers pri-miR482b-R and pri-miR482b-F were used in the RACE of the 5’ and 3’ ends, respectively (Additional file 2). The 5’ and 3’ cDNA fragments obtained from RACE were cloned into a pMD19-T vector (Takara) and sequenced. The RNA secondary structure was predicted by the RNAfold program [55].

### 4. In vitro transcription and Dicer splicing assay of pri-miR482b

The transcription and splicing assay for the pri-miR482b in vitro were performed according to *Qi et al.* [56]. The DNA template of pri-miR482b was amplified by using T7 promoter anchored primers (Additional file 2). Resulting DNAs were used for in vitro transcription under the presence of Biotin-UTP according to the manufacturer’s protocol (Roche). For Dicer activity assay, RNAs were incubated with 10 µg of protein in 20 µL of reaction buffer containing 100 mM NaCl, 1 mM ATP, 0.2 mM GTP, 1.2 mM MgCl_2_, 25 mM creatine phosphate, 30 µg/mL creatine kinase, and 4 U RNase Inhibitor at room temperature for 10 min. RNAs were extracted, precipitated, and dissolved in water.

The RNAs were electrophoresed in 8 M Urea 10 % PAGE gel and then transferred to a Hybond N + membrane (Amersham). The membrane was cross-linked twice with 254 nm UV light at 120 mJ/cm^2^. Biotin signal was detected by a Chemiluminescent Biotin-labeled Nucleic Acid Detection Kit (Beyotime, China) according to the manufacturer’s protocol. Images of the membranes were captured with a chemiluminescence detection system (Chemi-Doc; Bio-Rad Laboratories, Ltd., Hemel Hempstead, UK) with a CCD camera after 10 s of exposure.

### 5. Gene constructs and the generation of transgenic ***Arabidopsis*** plants

Two isoforms of pri-miR482b were amplified from the cDNA of tomato leaves using specific primers (Additional file 2) and then cloned into the pBIN438 expression vector downstream of the CaMV 35 S promoter region through the *Pst* I and *Xba* I restriction sites. The construct was introduced into *A. tumefaciens* GV3101 and transformed into *Arabidopsis “*Columbia” (Col-0) according to Wu et al. [53].

Methods and plant material described above complied with relevant institutional, national and international guidelines and legislation.

## Supplementary information

**Additional file 1**

**Additional file 2**

## Data Availability

All data generated during this study are included in this published article and its supplementary information files, except for the two isoform sequences of pri-miR482b which had been deposited into GenBank database (accession numbers: MW590251 and MW590252).

## Abbreviations

miRNAs: microRNAs
DCL1: Dicer-like 1
NBS-LRR: Nucleotide binding site-leucine-rich repeats
RISC: RNA-induced silencing complex
RT-qPCR: quantitative reverse transcription PCR
WT: wild type
OE: overexpression
RBPs: RNA-binding proteins
CaMV: Cauliflower mosaic virus
RACE: Rapid amplification of cDNA ends
PDF1.2: plant defensing 1.2
PR4: pathogen related protein 4
