# Supplementary material for: Molecular mechanism of modulating miR482b level in tomato with botrytis cinerea infection
Source: BMC Plant Biol. 2021 Oct 28;21:496. doi: 10.1186/s12870-021-03203-2 (PMC8555085; doi:10.1186/s12870-021-03203-2)
Supplement: Supplementary file 1 — Additional file 1 [file 12870_2021_3203_MOESM1_ESM.pdf]

Molecular Mechanism of Modulating MiR482b Level in Tomato with Botrytis Cinerea Infection

Fangli Wu, Jinfeng Xu, Tiantian Gao, Diao Huang, Weibo Jin*

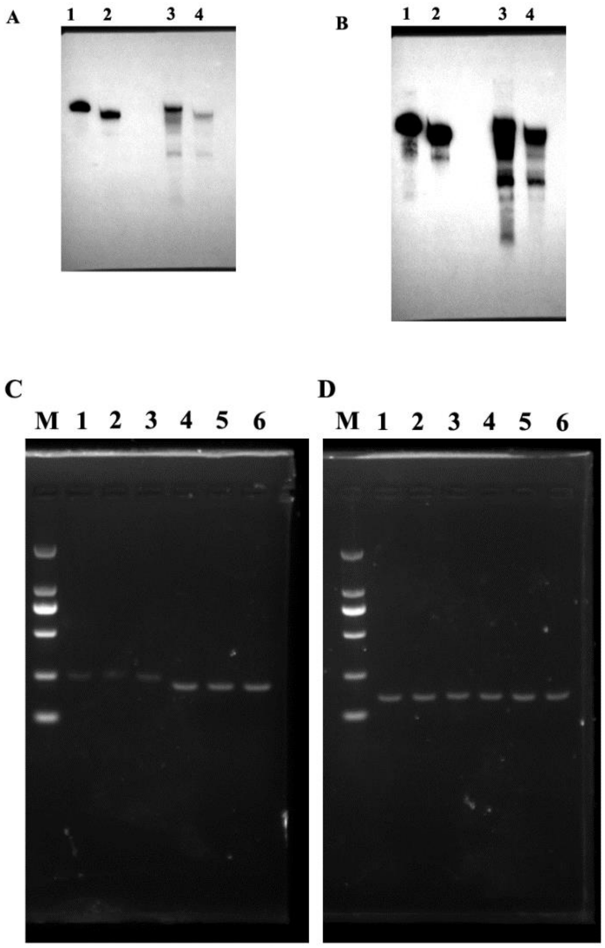

Figure S1: Original full-length gel and blot images.

A-B) The original blot image of Figure 4A with 10- (A) and 60-second (B) exposure time. Land 1: pri-miR482b-x1; Lane 2: pri-miR482b-x2; Lane 3: pri-miR482b-x1 with 2 mg/ml protein extraction; Lane 4: pri-miR482b-x2 with 2 mg/ml protein extraction. **C-D)** The original full-length gel image of Figure 4B for pri-miR482b-x1 and pri-miR482b-x2 (C) and *AtUBQ10* (D). Lane M: DL 2000 DNA marker; Lanes 1-3: three OE lines of pri-miR482b-x1 OE plants; Lanes 4-6: three OE lines of pri-miR482b-x2 OE plants.

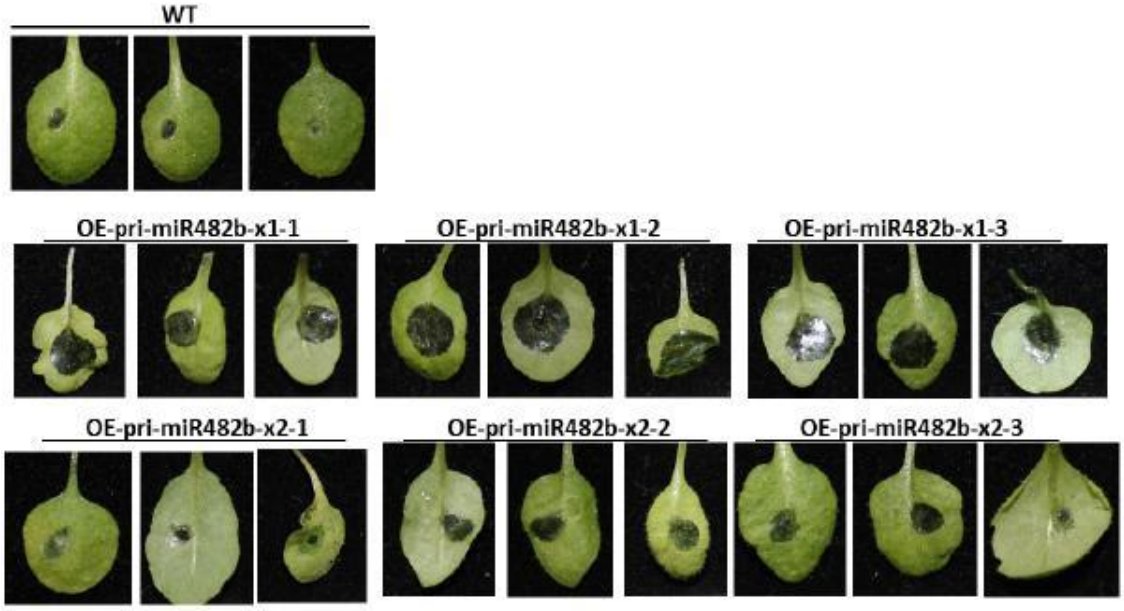

Figure S2: Resistance analysis of transgenic Arabidopsis overexpressing pri-miR482b-x1 and pri-miR482b-x2 against B. cinerea.
